# Measurement of cortisol in saliva: a comparison of measurement error within and between international academic-research laboratories

**DOI:** 10.1186/s13104-017-2805-4

**Published:** 2017-09-13

**Authors:** Jessica L. Calvi, Frances R. Chen, Victoria Brann Benson, Eleanor Brindle, Matt Bristow, Alpana De, Sonja Entringer, Helen Findlay, Christine Heim, Eric A. Hodges, Heiko Klawitter, Sonia Lupien, Holly M. Rus, Jitske Tiemensma, Silvanna Verlezza, Claire-Dominique Walker, Douglas A. Granger

**Affiliations:** 1 Salivary Bioscience Laboratory, University of Nebraska-Lincoln, Lincoln, NE 68588 USA; 2 Department of Criminal Justice and Criminology, Georgia State University, Atlanta, GA 30302 USA; 3 Biobehavioral Laboratory, The University of North Carolina at Chapel Hill, Chapel Hill, NC 27599-7460 USA; 4 Center for Studies in Demography and Ecology, University of Washington, Seattle, WA 98195 USA; 5 Department of Psychology, Anglia Ruskin University, Cambridge, CB1 1PT UK; 6 Department of Medical Psychology, Charité Universitätsmedizin, 10117 Berlin, Germany; 7 Development, Health, and Disease Research Program, University of California, Irvine, Irvine, CA 92868 USA; 8 Department of Biobehavioral Health, Pennsylvania State University, State College, PA 16802 USA; 9 Centre for Studies on Human Stress, Montreal Mental Health University Institute, Montreal, QC H1N 3V2 Canada; 10 Psychological Sciences, University of California, Merced, CA 95343 USA; 11 The Douglas Hospital Research Centre, McGill University, Montreal, QC H4H 1R3 Canada; 12 Bloomberg School of Public Health, The Johns Hopkins University, Baltimore, MD 21218 USA; 13 School of Nursing, The Johns Hopkins University, Baltimore, MD 21218 USA; 14 School of Medicine, The Johns Hopkins University, Baltimore, MD 21218 USA; 15 Institute for Interdisciplinary Salivary Bioscience Research, University of California, Irvine, Irvine, CA 92697-7085 USA

**Keywords:** Salivary cortisol, Inter-assay, Intra-assay, Inter-laboratory variation

## Abstract

**Objective:**

Hundreds of scientific publications are produced annually that involve the measurement of cortisol in saliva. Intra- and inter-laboratory variation in salivary cortisol results has the potential to contribute to cross-study inconsistencies in findings, and the perception that salivary cortisol results are unreliable. This study rigorously estimates sources of measurement variability in the assay of salivary cortisol within and between established international academic-based laboratories that specialize in saliva analyses. One hundred young adults (*Mean age*: 23.10 years; 62 females) donated 2 mL of whole saliva by passive drool. Each sample was split into multiple- 100 µL aliquots and immediately frozen. One aliquot of each of the 100 participants’ saliva was transported to academic laboratories (N = 9) in the United States, Canada, UK, and Germany and assayed for cortisol by the same commercially available immunoassay.

**Results:**

1.76% of the variance in salivary cortisol levels was attributable to differences between duplicate assays of the same sample within laboratories, 7.93% of the variance was associated with differences between laboratories, and 90.31% to differences between samples. In *established*-*qualified* laboratories, measurement error of salivary cortisol is minimal, and inter-laboratory differences in measurement are unlikely to have a major influence on the determined values.

## Introduction

Technical advances that have enabled salivary cortisol measurement have revolutionized research on sources of inter- and intra-individual differences in the reactivity and regulation of the hypothalamic–pituitary–adrenal (HPA) axis. Since the 1980s, thousands of publications have integrated salivary cortisol measurement across multiple disciplines and species. One pattern of note in the research literature, however, is that the substantive findings have not always been consistent across studies or between laboratories. Several possible explanations include variation between laboratories in saliva sample collection [1], sample integrity [2], participant noncompliance for collection timing [3], the number/frequency of samplings [4], specimen handling/transport [5], metrics applied to represent HPA axis (re)activity [6], and various analytic strategies [7–11]. Here we rigorously examine an alternative possibility—that variation in the performance of assays between laboratories contributes unsystematic error in salivary cortisol measurements. Accurate measures of cortisol levels assessed in saliva are important because they constitute the elementary components in the computation of several indices (area under the curve, cortisol awakening response, cortisol diurnal slope, latent trait cortisol) that are widely applied in psychopathology research to operationalize individual differences in the activity of the HPA axis.

## Main text

The first generation of salivary cortisol assays (predominantly pre-1990s), were idiosyncratic off-label, in-house modifications of commercially available serum-based immunoassays. In the late 1990s, enzyme immunoassays made specifically for saliva became commercially available. These assays substantially improved the lower limits of sensitivity, reduced (eight to tenfold) the saliva test volumes, eliminated the need to use substances in the mouth to stimulate saliva flow [12], and enabled “standardization” of assay protocols across laboratories.

Common metrics used to determine whether immunoassay results are *reliable and precise* include intra-assay precision (i.e., comparisons between results of the same sample assayed in duplicate) and *inter*-*assay precision* (i.e., comparison of results across assay runs). The scientific community recommendations [13, 14] provide guidelines that, on average, intra- and inter-assay precision, represented by the coefficient of variation (CV), should be less than 10 and 15%, respectively.

Multiple metrics are also used to determine whether an immunoassay is *valid*. Within each salivary cortisol assay, multiple *standards* (used to create a *standard curve*) and *controls* (i.e., samples with externally validated concentrations) are tested. The standard curve is used to translate idiosyncratic measurement units from laboratory reading equipment (i.e. optical density) into standard concentration/volume units (e.g., µg/dL or nmol/L) to allow comparison of assay results between studies and laboratories. The inclusion of *controls* serves as a validity check for each assay’s standard curve and can be used to evaluate inter-assay precision.

Laboratories that perform diagnostic testing are required to participate in proficiency testing programs (Clinical Laboratory Information Act 1988). The goal of these programs is to evaluate whether the same unknown sample tested by different laboratories generate comparable results. These procedures (with few exceptions) are rarely applied with salivary cortisol in the context of academic research. If such programs do occur, they often include samples from an artificial saliva-like matrix, which may not be representative with respect to range of expected values, viscosity, turbidity, and particulate matter in actual saliva samples. The present study is the first (to the best of our knowledge) to rigorously estimate the percent error in the measurement of salivary cortisol that can be attributed to sources within and between laboratories.

## Methods

### Participants

Participants were recruited via announcements and social networks at a large Southwestern University in the United States who met specific requirements (i.e., no medication intake, no acute or chronic medical condition, no dental work or problem within past 24 h). After obtaining informed consent, all participants rinsed their mouths with water and waited 10 min prior to saliva donation. There were 100 healthy young adults (*M* age = 23.10 years; 62 females; 69 Asian/Pacific Islanders, 28 European Americans, 3 multiracial) enrolled who each donated approximately 2 mL of whole saliva by passive drool. Each sample was split into multiple- 100 µL aliquots and immediately frozen; one aliquot of each of the 100 participants’ saliva was transported overnight on dry ice to academic laboratories in the United States, Canada, United Kingdom, and Germany. No samples were subject to more than one single freeze–thaw cycle and all samples were maintained frozen at least −60 °C. All laboratories were required to assay the samples for cortisol in duplicate, using the same commercially available immunoassay using reagents from the same manufactured lot without modification to the manufacturer’s (Salimetrics, Carlsbad, CA) recommended protocol.

### Laboratories

Participating laboratories were required to have at least 3 years’ experience assaying cortisol in saliva, use calibrated precision pipettes or robotic liquid handling systems, use multi-channel pipettes to handle assay reagent, use an automated plate washer, and determine optical densities using a plate reader with appropriate data reduction software. We verified whether the selected laboratories followed said procedures by administering a questionnaire. Ten laboratories were contacted to participate, all agreed to participate; one was disqualified due to lack of calibrated liquid handling and plate washing equipment, and data reduction software.

### Analytical strategy

To assay 100 samples in duplicate, each laboratory performed three assay runs. Performance within each lab was evaluated using the intra-assay CV for unknowns and inter-assay CV for controls, with lower values of each indicating high consistency *within* each laboratory. Performance *between* laboratories on the unknowns was evaluated with intra-class correlations (ICCs). Higher values suggest similar values between laboratories for the same samples.

Mean values generated by each laboratory were compared and estimated the percent variance attributable to inter-laboratory and sample differences in the measurement of salivary cortisol. In addition to the inter-assay CV for evaluating assay performance across runs, variance attributable to run/plate level was partitioned with a three-level model. Importantly, the six standards and the high/low controls were identical across all runs/plates and across all laboratories. Values were nested, allowing examination of observed variance at run/plate level, at laboratory level, and at sample level by evaluating the variance in each of the 6 standards, 2 controls, and 100 unknowns. If the effect of run/plate within each lab were low, it would suggest the each laboratory performed consistently across the three plates.

Last, variance in cortisol levels determined in the unknown saliva samples attributable to duplicates, laboratories, and intrinsic differences between specimens were evaluated. The total variance of the unknown saliva samples can be partitioned with a three-level linear mixed model (without predictors) into variance across duplicates of the same samples within each laboratory, variances across the nine laboratories for the same samples, and variances across the 100 samples as seen below,

$$\mathop \sum \limits_{i = 1}^{100} \mathop \sum \limits_{j = 1}^{9} \mathop \sum \limits_{k = 1}^{2} (x_{ijk} - \mu )^{2} = \mathop \sum \limits_{i = 1}^{100} \mathop \sum \limits_{j = 1}^{9} \mathop \sum \limits_{k = 1}^{2} (x_{ijk} - x_{ij} )^{2} + \mathop \sum \limits_{i = 1}^{100} \mathop \sum \limits_{j = 1}^{9} (x_{ijk} - x_{i} )^{2} + \mathop \sum \limits_{i = 1}^{100} (x_{i} - \mu )^{2}$$

where $x_{ijk}$ represents the value of sample i determined by laboratory j with the kth duplicate, and μ is the grand mean of all samples. Due to the nesting nature of the data, cluster means were included to capture the variance at each nesting level. For example, $x_{\text{ij}}$ is the mean of the duplicates for sample i determined by laboratory j, and $x_{\text{i}}$ is the mean of sample i across nine laboratories. The percentage of variances contributable to each level was computed by diving the variance at each level by the total variance.

## Results and discussion

### Preliminary analyses

On average, the R-square (RSQ) for the standard curves reflecting the relationship between B/BO (i.e., the percent bound, or the optical density of each well, B, divided by the average optical density of the zero standard well) and cortisol concentration (nmol/L) was 0.9999 (*SD* = 0.0001). The mean value for the low control was 3.03 nmol/L (*SD* = 0.03; range from 2.21 to 3.31 nmol/L) and mean value for the high control was 29.25 nmol/L (*SD* = 2.21; range from 23.45 to 35.32 nmol/L).

### Inter-and intra-assay CVs: within laboratories

Intra-assay CVs (within lab, between duplicate assays of the same sample) were, on average, 6.20% (*SD* = 1.36%). The inter-assay CVs (within lab, between runs/plates) were, on average, 6.36% (*SD* = 2.83%; see Table 1 for individual laboratory results).

**Table 1:** Intra-assay and inter-assay CVs by laboratories

Laboratory	Intra-assay CV mean (standard deviation)	Inter-assay CV
A	6.70 (4.25)	4.49
B	5.09 (4.18)	4.11
C	4.82 (6.22)	9.94
D	7.75 (11.75)	5.68
E	5.81 (4.76)	3.57
F	8.54 (8.34)	11.14
G	6.82 (5.73)	3.90
H	4.57 (4.03)	5.80
I	5.66 (6.70)	8.65

### ICCs: between laboratories

**Fig. 1:**
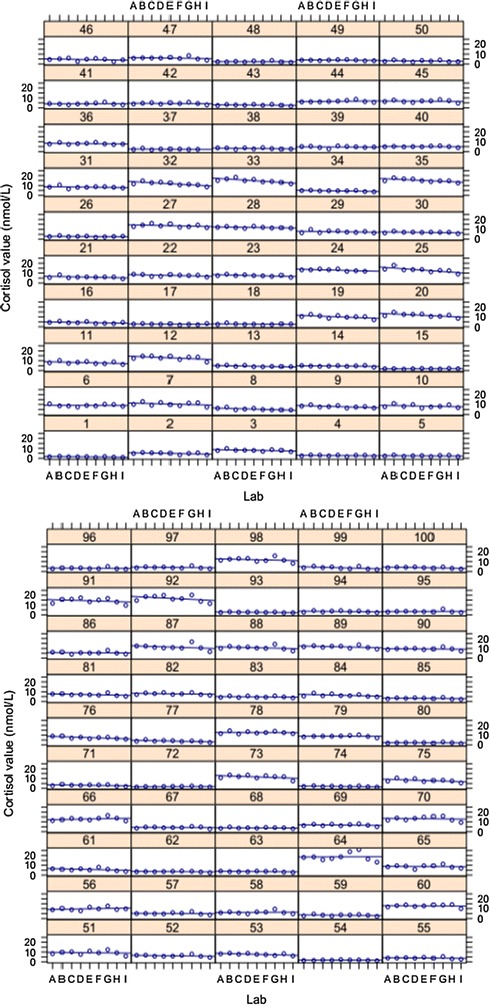
Variation in salivary cortisol levels across 9 laboratories on the same 100 samples. The x-axis represents the 9 labs labeled with letters. Each small panel with a number on top represents a sample that was assayed by nine different labs (blue dots)

The ICC was high (*r* = 0.91; 95% CI 0.88–0.93), suggesting a high degree of consistency between participating laboratories for each of the unknown cortisol values (see Fig. 1).

### Variance attributable to measurement errors in salivary cortisol

For all standards and controls, the values from the three plates are nested within labs, and then further nested in each of the six standards and controls. Thus, variance contributable to plates with a three-level model ca be partitioned. Only 0.11% of total variance in the standards and controls are attributable to run/plate differences (Table 2), indicating that the laboratories performed consistently across their three plates.

**Table 2:** Three-level model variance partition of standards and high/low controls across laboratories and plates

	9 laboratories (standards + 2 controls)	
Variance across plates for the same samples within laboratories	0.001	0.11%
Variance across laboratories for the same samples	0.001	0.11%
Variance across samples	0.894	99.78%
Total variance	0.896	

A separate three-level model was run on the unknown values, with duplicates nested in laboratories, and the nine laboratories nested in each of the 100 samples. Results revealed that 1.76% of the variance in cortisol determination was due to differences between duplicate assays, 7.93% due to differences between labs, and 90.31% due to differences between specimens.

Multi-level linear mixed modeling revealed that small amounts of the variance were attributable to differences between duplicate testing of the same sample and between different laboratories testing of the same samples (i.e., 1.76 and 7.93%, respectively). The precision and reproducibility of findings between duplicate tests, between runs/plates within any individual laboratory, and between labs is well within immunodiagnostic industry standards, [13, 14]. Participating laboratories were selected because they (1) follow well-established and well-executed standard operating procedures, (2) adhere to strict quality control metrics, (3) maintain calibrated liquid handling and reading equipment, and (4) maintain electronic records of quality control metrics to monitor assay performance over time. The probability that the present observations generalize to other laboratories that follow similar procedures is high.

The recommended guidelines to repeat assaying any sample was an absolute difference between duplicates greater than 0.83 nmol/L *and* a CV above 15%. Four laboratories adhered to this guideline and they each had 2, 1, 4, and 4 samples that required repeats. In two laboratories, none of their samples met the repeat criteria. Two reported their criterion for repeats was a CV over 15%, and they had 7 and 9 samples needed repeats. One laboratory did not conduct any repeat or report their guideline for such practice, and they had 4 samples that meet the recommended repeat guideline. In the current statistical analyses, only initial analysis of the saliva samples (not the repeats) were used. This represents a “worst case scenario”, namely a one-shot analysis of saliva samples for cortisol. When adhering to the repeat guideline, the variance attributable to measurement error and inter-laboratories would only diminish.

## Concluding comment

To the best of our knowledge, there has been no other study of the reproducibility of results in salivary cortisol analysis across academic laboratories. Concerns about the reproducibility of findings has emerged in the recent past in multiple disciplines and subfields of scientific inquiry. Here we have taken a step to confirm the reproducibility and precision of the measurement of salivary cortisol. Our conclusion is that in *established*-*qualified* laboratories, measurement error of salivary cortisol is minimal, and inter-laboratory differences in measurement are unlikely to have a major influence on the determined values. We encourage researchers to establish their own evidence of reproducibility and consistency across laboratory analyses by outlining standard procedures, including uniform training for laboratory personnel, preventative care and maintenance of laboratory equipment, adopting uniform methods for repeat criteria of salivary cortisol samples, and maintaining high standards for both intra-assay and inter-assay coefficients of variation (<10 and <15%, respectively).

## Limitations

On the other hand, the probability that these observations would generalize to laboratories that lack the quality standards noted above is highly questionable. A limitation of the present study is that research design prevents us from addressing this possibility directly. It is tempting to speculate that laboratories that have contributed salivary cortisol data to the published literature that do not fit the quality profile above have contributed to cross-study inconsistencies in salivary cortisol findings, or have had limited capacity to detect cortisol effects/relationships, or both.

## Abbreviations

HPA: hypothalamic–pituitary–adrenal
CV: coefficient of variation
ICC: intra-class correlation
RSQ: R-square
B**/**Bo: percent bound

## References

[1] Granger DA, Kivlighan KT, Fortunato C, Harmon AG, Hibel LC, Schwartz EB (2007). Integration of salivary biomarkers into developmental and behaviorally-oriented research: problems and solutions for collecting specimens. Physiol Behav.

[2] Granger DA, Cicchetti D, Rogosch FA, Hibel LC, Teisl M, Flores E (2007). Blood contamination in children’s saliva: prevalence, stability, and impact on the measurement of salivary cortisol, testosterone, and dehydroepiandrosterone. Psychoneuroendocrinology.

[3] Stalder T, Kirschbaum C, Kudielka BM, Adam EK, Pruessner JC, Wüst S (2016). Assessment of the cortisol awakening response: expert consensus guidelines. Psychoneuroendocrinology.

[4] Adam EK, Kumari M (2009). Assessing salivary cortisol in large-scale, epidemiological research. Psychoneuroendocrinology.

[5] Whembolua GLS, Granger DA, Singer S, Kivlighan KT, Marguin JA (2006). Bacteria in the oral mucosa and its effects on the measurement of cortisol, dehydroepiandrosterone, and testosterone in saliva. Horm Behav.

[6] Granger DA, Fortunato CK, Beltzer EK, Virag M, Bright MA, Out D (2012). Focus on Methodology: salivary bioscience and research on adolescence: an integrated perspective. J Adolesc.

[7] Adam EK (2006). Transactions among adolescent trait and state emotion and diurnal and momentary cortisol activity in naturalistic settings. Psychoneuroendocrinology.

[8] Doane LD, Chen FR, Sladek MR, Van Lenten SA, Granger DA (2015). Latent trait cortisol (LTC) levels: reliability, validity, and stability. Psychoneuroendocrinology.

[9] Pruessner JC, Gaab J, Hellhammer DH, Lintz D, Schommer N, Kirschbaum C (1997). Increasing correlations between personality traits and cortisol stress responses obtained by data aggregation. Psychoneuroendocrinology.

[10] Pruessner JC, Kirschbaum C, Meinlschmid G, Hellhammer DH (2003). Two formulas for computation of the area under the curve represent measures of total hormone concentration versus time-dependent change. Psychoneuroendocrinology.

[11] Willoughby M, Vandergrift N, Blair C, Granger DA (2007). A structural equation modeling approach for the analysis of cortisol data collected using pre–post–post designs. Struct Equ Model A Multidiscip J.

[12] Schwartz EB, Granger DA, Susman EJ, Gunnar MR, Laird B (1998). Assessing salivary cortisol in studies of child development. Child Dev.

[13] Chard T (1995). An introduction to radioimmunoassay and related techniques.

[14] Wild D (2013). The Immunoassay Handbook: theory and applications of ligand binding. ELISA Relat Tech.

